# On Stability of Continuous Cooperative Static Games with Possibilistic Parameters in the Objective Functions

**DOI:** 10.1155/2022/6979075

**Published:** 2022-05-06

**Authors:** Harish Garg, S. A. Edalatpanah, Salwa El-Morsy, Hamiden Abd El- Wahed Khalifa

**Affiliations:** ^1^School of Mathematics, Thapar Institute of Engineering & Technology, Deemed University, Patiala 147004, Punjab, India; ^2^Department of Applied Mathematics, Ayandegan Institute of Higher Education, Tonekabon, Iran; ^3^Basic Science Department, Nile Higher Institute for Engineering and Technology, Mansoura 35511, Egypt; ^4^Department of Mathematics, College of Science and Arts, Qassim University, Al- Badaya 51951, Saudi Arabia; ^5^Department of Operations Research, Faculty of Graduate Studies for Statistical Research, Cairo University, Giza 12613, Egypt

## Abstract

The objective of this paper is to present a novel idea about the continuous possibilistic cooperative static game (Poss-CCSTG). The proposed Poss-CCSTG is a continuous cooperative static game (CCSTG) in which parameter associated with the cost functions of the players involves the possibility measures. The considered Poss-CCSTG is converted into the crisp *α*-CCSTG problem by using the *α*-cuts and hence into the multiple objective nonlinear programming problem. To solve the formulated *α*-CCSTG problem, an interactive approach is presented in the study with the use of the reference direction method. Further, the Lexicographic weighted Tchebycheff model is derived to obtain the weights. Also, a parametric study corresponding to the *α*-possibly optimal solution is defined and determined. Finally, a decision-maker can compare their desired solution with the attainable reference point and the weak efficient solution. The presented model is illustrated with a numerical example and its advantages are stated.

## 1. Introduction

Game theory is one of the essential theories in optimization techniques. It plays a vital role in many engineering fields such as Economics, Engineering, Biology, computational engineering, and other mathematical sciences with many applications in real-world problems [1]. The most crucial types of games are differential games, matrix games, and continuous static games. Matrix games derive their name from a discrete relationship between a finite/countable number of possible decisions and the related costs. The connection is conveniently represented in a matrix (or two-player games) where one player's decision corresponds to the selection of a row and the other player's decision to select a column, with the corresponding entries denoting the costs. It is intense that decision probabilities are not mandatory in cooperative games. In addition, there is no time in the relationship between costs and decisions in static games. Differential games are identified by continuously varying costs and a dynamic system controlled by ordinary differential equations. For continuous static games, there are several solution concepts. How players use these concepts depends not only on information concerning the nature of the other players but also on their personality. A given player may or may not play rationally, cheat, cooperate, bargain, and so on. A player making the ultimate choice of their control vector must consider all of these factors. Thomas and Walter [2] introduced different formulations in continuous static games. The three basic solution concepts for these games are as follows:

1. Nash equilibrium solution
2. Min-max solutions
3. Pareto minimal solutions

In our day-to-day life, uncertainties play a dominant role and occur almost in each sector. To handle it, Zadeh [3] stated the innovative concept of fuzzy set which relates every component of the universal set to a unique real number, called membership degree. By using a fuzzification principle, Dubois and Prade [4] expanded the applications of algebraic operations on real numbers to fuzzy numbers. Bellman and Zadeh [5] developed decision-making in a fuzzy environment, which improved the management decision problems. Kaufmann and Gupta [6] worked on various fuzzy mathematical models with their applications in management sciences and engineering. Ebrahimnejad [7] presented an algorithm for solving the fully fuzzy linear programming problems. Osman [8] formulated different parametric problems of continuous static games. Osman et al. proposed Stackelberg leader with min-max follower's solution [9] to solve continuous static games with fuzzy parameters and introduced the parametric analysis for the solution. For large scale, continuous static games with parameters in all cost functions and limitations, Osman et al. [10] developed the Nash equilibrium solution. In this type of game, the players are independent without participation with any other players, and every player seeks to minimize their cost functions. In addition, the information that is available to every player contains the cost functions and constraints. To solve Nash Cooperative Continuous Static Games, Elshafei [11] established an interactive approach and fixed on the first-kind corresponding's stability set to the obtained compromise solution. Several articles were developed for the game's theory by an enormous of research, and for more details about such studies, we refer to read the articles mentioned in [12–21]. Shuler [22] looked at cooperative games in which the impoverished agents profiting from collaboration with the wealthy is not applicable. Khalifa and Kumar [23] studied the cooperative continuous static games in a crisp environment, defined them, and found the first-kind stability set corresponding to the solution without differentiability. Several researchers [24–29] have recently enriched the theory of cooperative games by considering the degree of uncertainties in the analysis.

The present work studies the concept of continuous static games (CSG) under an uncertain environment by keeping the above literature in mind. For this, CSG is considered under the possibilistic environment in the study. In this game, it is supposed that every player helps the others up to the point of disadvantages to himself. To discuss it in detail, a concept of Pareto optimal solution is discussed in which cooperation between all of the players is taken into account. To handle the uncertainties in the game theory, a concept of the continuous possibilistic cooperative static game (Poss-CCSTG) is proposed. The proposed Poss-CCSTG is a continuous cooperative static game (CCSTG) in which the cost functions associated with *m* players involve the possibility measures. By using the concept of the *α*-cuts, the considered Poss-CCSTG is converted into the crisp *α*-CCSTG problem and hence into the multiple objective nonlinear programming problem. To solve the formulated *α*-CCSTG problem, an interactive approach is presented in the study with the use of the reference direction method. Furthermore, the *α*-Parametric efficient solution for players' cooperation is discussed in the study. The main objective of the study is considered as

1. An idea related to the CCSTG with possibilistic parameters associated with the cost functions of the *m* players is discussed. To handle the uncertainties in the model and a conflicting nature between the objectives, a possibilistic variable ${\tilde{a}}_{i}$ which is categorized by a possibilistic distribution $\mu_{{\tilde{a}}_{i}}:V⟶\left[0,1\right]$ for *i*=1,2,…, *n* is taken in the study.
2. *α*-possibly efficient solution, *α*-parametric efficient solution, and the relationship between them for a Poss-CCSTG are characterized.
3. An interactive algorithm is presented to solve the Poss-CCSTG by considering the decision-maker's preference.
4. The first-kind stability set that corresponds to the solution is conceptuaized.
5. A numerical example is provided to validate the proposed study. Also, a characteristic comparison of the proposed study over several other existing studies is examined.

**Figure 1:**
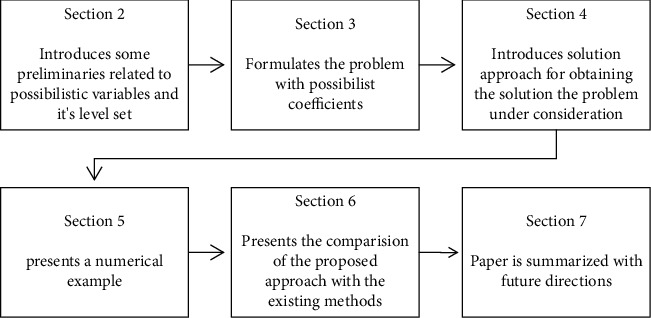
Structure of the paper.

The organization of the article is summarized in Figure 1.

## 2. Preliminaries

In this section, we recall the basic definitions related to possibilistic variables and their properties. Let V be a universal set.

**Definition 1 (see [30, 31]).**

A possibilistic variable *Z* on *V* is a variable categorized by a possibility distribution *μ*_*z*_(*v*) : *V*⟶[0,1].

In other words, we can say that if *v* is a variable taking values in *V*, then *μ*_*z*_ corresponding to *z* may be viewed as a fuzzy constraint. Such a distribution is characterized by a possibility distribution function *μ*_*z*_ which is associated with each *v* ∈ *V*, the degree of compatibility of *z* with the realization *v* ∈ *V*.

**Remark 1 .**

If *V* is a Cartesian product of *V*_1_,  *V*_2_,…,  *V*_*n*_, then *μ*_*z*_(*v*_1 _, *v*_2_,…, *v*_*n*_) is an *n*−ary possibility distribution, i.e., *μ*_*z*_(*v*)=(*μ*_*z*_1__(*v*_1_), *μ*_*z*_2__(*v*_2_),…, *μ*_*z*_*n*__(*v*_*n*_)).

**Definition 2 (see [30]).**

The *α*-level set of possibilistic variable *Z* is defined as

(1) $$\begin{array}{c} Z_{\alpha }=\left\{v\in V:\mu_{z}\left(v\right)\geq \alpha \right\}. \end{array}$$

**Definition 3 (see [30]).**

A possibility distribution *μ*_*z*_ on *V* is said to be convex if

(2) $$\begin{array}{c} \mu_{z}\left(\gamma v^{1}+\left(1-\gamma \right)v^{2}\right)\geq \min\left(\mu_{z}\left(v^{1}\right),\mu_{z}\left(v^{2}\right)\right);\forall v^{1},v^{2}\in V,\gamma \in \left[0,1\right]. \end{array}$$

**Definition 4 (see [31]).**

The support of a possibilistic variable *Z* is defined as

(3) $$\begin{array}{c} \text{Supp}\left(Z\right)=\left\{v\in V:\underset{u\in N_{\varepsilon }\left(u\right)}{\text{Sup}}\left(\mu_{z}\left(u\right)\right)>0;\forall \varepsilon >0\right\}, \end{array}$$

where *N*_*ε*_(*v*)={*v* ∈ *V* : *u* − *v* < *ε*}.

**Remark 2 (see [30]).**

Supp(*Z*) is closed set on *V*.

## 3. Formulation of the Problem with Possibilistic Variables

In this section, we present the concept of the Poss-CCSTG with *m* players. Consider a game problem with *m* players having possibilistic parameters in the cost functions as

(4) $$\begin{array}{c} G_{1}\left(b,\xi ,{\tilde{a}}_{1}\right),G_{2}\left(b,\xi ,{\tilde{a}}_{2}\right),\ldots ,G_{m}\left(b,\xi ,{\tilde{a}}_{m}\right), \end{array}$$

(5) $$\begin{array}{c} g_{j}\left(b,\xi \right)=0,j=\bar{1,n}, \end{array}$$

(6) $$\begin{array}{c} \xi \in \Psi =\left\{\xi \in {\mathbb{R}}^{t}:h_{l}\left(b,\xi \right)\geq 0,l=1,2,\ldots ,s\right\}, \end{array}$$

where $G_{i}\left(b,\xi ,{\tilde{a}}_{i}\right),i=\bar{1,m}$, are convex functions on *ℝ*^*m*^ × *ℝ*^*t*^, $h_{l}\left(b,\xi \right),l=\bar{1,s}$, are concave functions on ${\mathbb{R}}^{n}\times {\mathbb{R}}^{t};g_{j}\left(b,\xi \right),j=\bar{1,m}\;$, stated as *j*=1,2,…, *m* are convex functions on ℝ^*n*^ × ℝ^*t*^, and *a* is a possibilistic *n*−ary, i.e., ${\tilde{a}}_{i},i=\bar{1,n}$, are possibilistic variables on ℝ^*n*^ characterized by the possibilistic distributions $\mu_{{\tilde{a}}_{i}}$ (Luhandjula [32, 33]). If the functions $g_{j}\left(b,\xi \right),j=\bar{1,m}$ are differentiable, then the Jacobian $\left|\delta g_{j}\left(b,\;\xi \right)/\delta b_{k}\right|\neq 0,j,k=\bar{1,n}$, in a neighborhood of a solution point (*b*, *ξ*) to (2). *b*=*f*(*ξ*) is the solution to (2) generated by *ξ* ∈ Ψ. It is noted her that the differentiability assumptions are not needed for the functions *G*_*i*_(*b*, *ξ*, *a*_*i*_) and *h*_*l*_(*b*, *ξ*). Ψ is a regular and compact set.

The considered Poss-CCSTG model defined in equations (4)–(6) transforms into *α*-CCSTG based on a certain degree *α* ∈ [0,1] as

(7) $$\begin{array}{c} \left(\alpha -CCSTG\right)G_{1}\left(b,\xi ,a_{1}\right),G_{2}\left(b,\xi ,a_{2}\right),\ldots ,G_{m}\left(b,\xi ,a_{m}\right)\\ g_{j}\left(b,\xi \right)=0,j=\bar{1,n},\\ \xi \in \Psi ,a_{i}\in u_{\alpha }\left({\tilde{a}}_{i}\right),i=\bar{1,m}. \end{array}$$

Here, it should be noted that in problem (7), the parameters ${\tilde{a}}_{i},i=1,\;2,\ldots ,m$, are decision variables not constants.

**Definition 5 .**

Let *b*=*f*(*ξ*) denote the solution to (5) generated by *ξ* ∈ Ψ. A point *ξ*^*∗*^ ∈ Ψ is said to be *α*−possibly efficient solution to the *α* − CCSTG (4) if and only if there does not exist $\left(\xi ,a\right)\in \Psi \times u_{\alpha }\left({\tilde{a}}_{i}\right)$ such that

(8) $$\begin{array}{c} G_{i}\left(f\left(\xi \right),\xi ,a_{i}\right)\leq G_{i}\left(f\left(\xi \right),\xi^{*},a_{i}{\;}^{*}\right);\forall i\in \left\{1,2,\ldots ,m\right\}. \end{array}$$

*G*_*i*_(*f*(*ξ*), *ξ*, *a*_*i*_) < *G*_*i*_(*f*(*ξ*), *ξ*^*∗*^, *a*_*i*_ ^*∗*^); ∀*i* ∈ {1,2,…, *m*} for some *j* ∈ {1,2,…, *n*}where *a*_*i*_ ^*∗*^ are *α*−level minimal parameters.

From the concept of *α*−possibly efficient solution to the *α* − CCSTG, one can see that *ξ*^*∗*^ ∈ Ψ is an *α*−possibly efficient solution to the problem (7), if and only if *ξ*^*∗*^ is an *α*− parametric efficient solution to the following *α*− possibilistic multiobjective nonlinear programming (*α* − P MONLP) problem (Vincent and Grantham [2]).

(9) $$\begin{array}{c} \left(\alpha -\text{PMONLP}\right)\min{\left(G_{1}\left(\xi ,a_{1}\right),G_{2}\left(\xi ,a_{2}\right),\ldots ,G_{m}\left(\xi ,a_{m}\right)\right)}^{T},\\ \xi \in \Psi ;a_{i}\in u_{\alpha }\left({\tilde{a}}_{i}\right),i=\bar{1,m}, \end{array}$$

where *G*_*i*_(*ξ*, *a*_*i*_), *i*=1,…, *n*, are convex functions on ℝ^*t*^ × ℝ^*n*^ and ${\dddot{h}}_{l}\left(\xi \right),l=1,\ldots ,s$, are concave functions on ℝ^*t*^, and $G_{i}\left(\xi ,a_{i}\right)=G_{i}\left(f\left(\xi \right),\xi ,a_{i}\right),{\dddot{h}}_{l}\left(\xi \right)={\overset{\ldots }{h}}_{l}\left(f\left(\xi \right),\xi \right)$. $u_{\alpha }\left({\tilde{a}}_{i}\right)$ is the *α*− cut of ${\tilde{a}}_{i}$.

By the convexity assumption, $u_{\alpha }\left({\tilde{a}}_{i}\right),i=1,\ldots ,m$, are real intervals denoted by $\left[{\tilde{a}}_{i}{\;}^{L}\left(\alpha \right),{\tilde{a}}_{i}{\;}^{U}\left(\alpha \right)\;\right]$, $i=\bar{1,m}.$ Then, clearly, the *α* − PMONLP can be rewritten as follows:

(10) $$\begin{array}{c} \min G_{i}\left(\xi ,a_{i}\right),i=\bar{1,m}. \end{array}$$

(11) $$\begin{array}{c} \xi \in \Psi ;a_{i}\in \left[{\tilde{a}}_{i}{\;}^{L}\left(\alpha \right),{\tilde{a}}_{i}{\;}^{U}\left(\alpha \right)\right]=\varphi_{i\alpha },i=\bar{1,m}. \end{array}$$

**Definition 6 .**

*ξ* ^*∗*^ is an *α*−parametric efficient solution for *α* − PMONLP if and only if there is no *ξ* ∈ Ψ and *a*_*i*_ ∈ *φ*_*iα*_ such that $G_{i}\left(\xi ,a_{i}\right)\leq G_{i}\left(\xi^{*},a_{i}\right);\forall i=\bar{1,m}$ and *G*_*i*_(*ξ*^*∗*^, *a*_*i*_) < *G*_*i*_(*ξ*^*∗*^, *a*_*i*_) for at least one *i*.

**Theorem 1 .**

*ξ* ^*∗*^ ∈ Ψ is an *α*−possibly efficient solution for Poss-CCSTG if and only if *ξ*^*∗*^ ∈ Ψ is an *α*−parametric efficient solution for *α* − PMONLP.

**Proof**

Necessity.

Let *ξ*^*∗*^ ∈ Ψ be an *α*−possibly efficient solution for Poss-CCSTG and *ξ*^*∗*^ ∈ Ψ be not *α*−parametric efficient solution for *α* − PMONLP. Then, there are *ξ*^1^ ∈ Ψ and *d*_*i*_ ∈ *φ*_*iα*_, *i* = 1,2,…, *m*, such that

(12) $$\begin{array}{c} G_{r}\left(\xi^{1},d_{r}\right)\leq G_{r}\left(\xi^{*},d_{r}\right);\forall r,i\in \left\{1,\ldots ,m\right\}. \end{array}$$

As *a*_*i*_ ∈ *φ*_*iα*_, *i*=1,…, *m*, we get Poss $\left(\begin{array}{c} G_{1}\left(\xi^{1},{\tilde{a}}_{1}\right)\leq G_{1}\left(\;\xi^{*},{\tilde{a}}_{1}\right),\ldots ,G_{i-1}\left(\xi^{1},{\tilde{a}}_{i-1}\right)\leq G_{i-1}\left(\;\xi^{*},{\tilde{a}}_{i-1}\right),\\ G_{i}\left(\xi^{1},{\tilde{a}}_{i}\right)\leq G_{I}\left(\;\xi^{*},{\tilde{a}}_{I}\right),\;G_{i+1}\left(\xi^{1},{\tilde{a}}_{i+1}\right)\leq G_{i+1}\left(\;\xi^{*},{\tilde{a}}_{i+1}\right),\\ \ldots ,G_{m}\left(\xi^{1},{\tilde{a}}_{m}\right)\leq G_{m}\left(\;\xi^{*},{\tilde{a}}_{m}\right) \end{array}\right)\geq \alpha$.

This contradicts the *α*−possibly efficient solution of Poss-CCSTG.

Let  *ξ*^*∗*^ ∈ Ψ be an *α*−parametric efficient solution for *α* − PMONLP and  *ξ*^*∗*^ ∈ Ψ be not *α*−possibly efficient solution of Poss-CCSTG. Then, there are *ξ*^2^ ∈ Ψ and *i* ∈ {1,…,  *m*} such that Poss $\left(\begin{array}{c} {\text{G}}_{1}\left(\xi^{2},{\tilde{\text{a}}}_{1}\right)\leq {\text{G}}_{1}\left({\;\xi }^{*},{\tilde{\text{a}}}_{1}\right),\ldots ,{\text{G}}_{\text{i}-1}\left(\xi^{2}{\tilde{\text{a}}}_{\text{i}-1}\right)\leq {\text{G}}_{\text{i}-1}\left({\;\xi }^{*},{\tilde{\text{a}}}_{\text{i}-1}\right),\\ {\text{G}}_{\text{i}}\left(\xi^{2},{\tilde{\text{a}}}_{\text{i}}\right)\leq {\text{G}}_{\text{I}}\left({\;\xi }^{*},{\tilde{\text{a}}}_{\text{I}}\right),\;{\text{G}}_{\text{i}+1}\left(\xi^{2},{\tilde{\text{a}}}_{\text{i}+1}\right)\leq {\text{G}}_{\text{i}+1}\left({\;\xi }^{*},{\tilde{\text{a}}}_{\text{i}+1}\right),\\ \ldots ,{\text{G}}_{\text{m}}\left(\xi^{2},{\tilde{\text{a}}}_{\text{m}}\right)\leq {\text{G}}_{\text{m}}\left({\;\xi }^{*},{\tilde{\text{a}}}_{\text{m}}\right) \end{array}\right)\geq \alpha$.

(13) $$\begin{array}{c} \underset{\left(a_{1},\;\ldots ,\;a_{m}\right)\in \bar{A}}{\sup}\min\left(\mu_{{\tilde{a}}_{1}}\left(a_{1}\right),\;\mu_{{\tilde{a}}_{2}}\left(a_{2}\right),\ldots ,\mu_{{\tilde{a}}_{m}}\left(a_{m}\right)\right)\geq \alpha , \end{array}$$

where

(14) $$\begin{array}{c} \bar{A}=\left\{\begin{array}{c} \left(a_{1},\;\ldots ,\;a_{n}\right)\in {\mathbb{R}}^{n}:\;G_{1}\left(\xi^{2},{\tilde{a}}_{1}\right)\leq G_{1}\left(\;\xi^{*},{\tilde{a}}_{1}\right),\ldots ,\\ G_{i-1}\left(\xi^{2},{\tilde{a}}_{i-1}\right)\leq G_{i-1}\left(\;\xi^{*},{\tilde{a}}_{i-1}\right),G_{i}\left(\xi^{2},{\tilde{a}}_{i}\right)\leq G_{i}\left(\;\xi^{*},{\tilde{a}}_{i}\right),\\ G_{i+1}\left(\xi^{2},{\tilde{a}}_{i+1}\right)\leq G_{i+1}\left(\;\xi^{*},{\tilde{a}}_{i+1}\right),\ldots ,G_{m}\left(\xi^{2},{\tilde{a}}_{m}\right)\leq G_{m}\left(\;\xi^{*},{\tilde{a}}_{m}\right) \end{array}\right\}. \end{array}$$

For the supremum to be exist, there is $\left({\text{e}}_{1},{\text{ e}}_{2},\;\ldots ,{\text{e}}_{\text{m}}\right)\in \bar{\text{A}}$ with $\min\left(\mu_{{\tilde{\text{a}}}_{1}}\left({\text{e}}_{1}\right),\mu_{{\tilde{\text{a}}}_{2}}\left(\text{e}\right),\ldots ,\mu_{{\tilde{\text{a}}}_{\text{m}}}\left({\text{e}}_{\text{m}}\right)\right)<\alpha$, then

(15) $$\begin{array}{c} \underset{\left(e_{1},e_{2},\;\ldots ,e_{m}\right)\in \bar{A}}{\sup}\min\left(\mu_{{\tilde{a}}_{1}}\left(e_{1}\right),\mu_{{\tilde{a}}_{2}}\left(e\right),\ldots ,\mu_{{\tilde{a}}_{m}}\left(e_{m}\right)\right)<\alpha . \end{array}$$

This contradicts equation (6).

Hence, there is $\left(e_{1},e_{2},\;\ldots ,e_{m}\right)\in \bar{A}$ satisfying

(16) $$\begin{array}{c} \min\left(\mu_{{\tilde{a}}_{1}}\left(e_{1}\right),\mu_{{\tilde{a}}_{2}}\left(e\right),\ldots ,\mu_{{\tilde{a}}_{m}}\left(e_{m}\right)\right)\geq \alpha , \end{array}$$

(17) $$\begin{array}{c} e_{i}\in \varphi_{i\alpha },\;i=\bar{1,\;m}. \end{array}$$

From (16) and (17), we conclude to the contradiction that  *ξ*^*∗*^ is an *α*−parametric efficient solution for *α* − PMONLP.

## 4. Solution Approach

This section stated the interactive solution procedure for solving the above formulated possibilistic models. The steps of the proposed approach are summarized as follows:

*Step 1.* Formulate the (*α* − MONLP) problem, after the decision-maker specifies the initial value of *α*(0 < *α* < 1).
*Step 2*. Solve the following problem:
  (18) $$\begin{array}{c} \underset{\text{i}=1,\;2,\;\ldots ,\text{ n}}{\max}{\text{w}}_{\text{i}},\\ {\text{G}}_{\text{i}}\left(\xi ,{\text{a}}_{\text{i}}\right)\geq {\text{w}}_{\text{i}},\text{i}=\bar{1,\text{m}},\\ \xi \in \Psi =\left\{\xi \in {\mathbb{R}}^{\text{t}}:{\overset{\ldots }{\text{h}}}_{\text{l}}\left(\xi \right)\geq 0,\text{l}=\bar{1,\text{s}}\right\},\\ {\text{a}}_{\text{i}}\in {\text{u}}_{\alpha }\left({\tilde{\text{a}}}_{\text{i}}\right),\text{i}=\bar{1,\text{m}};{\text{w}}_{\text{i}}\in \mathbb{R}. \end{array}$$
After solving this model, assume $\overset{ˇ}{\text{G}}$ be its optimum value.
*Step 3*. Given initial reference point.
Decision-maker provides an initial attainable reference point ${\bar{\text{G}}}^{0}$ such that ${\bar{\text{G}}}^{0}>\overset{ˇ}{\text{G}}$.
Let I={1,  2,…, m}, I^0^=I, k=0.
*Step 4*. Search for a reference *α*−possibly efficient solution.
Let ${\bar{y}}_{i}={\bar{G}}_{i}^{k}-{\overset{ˇ}{G}}_{i}/\sum_{i=1}^{n}\left({\bar{G}}_{i}^{k}-{\overset{ˇ}{G}}_{i}\right),i=\bar{1,m}.$ Then, consider the following Lexicographic weighted Tchebycheff program (LEWT):
  (19) $$\begin{array}{c} \left(\text{LEWT}\right)\text{Lex }\underset{i=1,2,\;\ldots ,m}{\min}\left\{\gamma_{i},\sum_{i=1}^{n}\left|G_{i}\left(\xi ,a_{i}\right)-{\overset{ˇ}{G}}_{i}\right|\right\}, \end{array}$$
  (20) $$\begin{array}{c} {\bar{y}}_{i}\left|G_{i}\left(\xi ,\;a_{i}\right)-{\overset{ˇ}{G}}_{i}\right|\leq \gamma_{\text{I}},\;i=\bar{1,\;n}, \end{array}$$
  (21) $$\begin{array}{c} \xi \in \Psi =\left\{\xi \in {\mathbb{R}}^{t}:{\overset{\ldots }{h}}_{l}\left(\xi \right)\geq 0,l=1,2,\ldots ,\;s\right\}, \end{array}$$
  (22) $$\begin{array}{c} \;a_{i}\in u_{\alpha }\left({\tilde{a}}_{i}\right),\;i=\bar{1,\;n};0\leq \gamma_{i}\in \mathbb{R}. \end{array}$$
After solving this LEWT model, we get the *α*−possibly optimal solution as (*ξ*^*k*^,  *a*_*i*_^*k*^).
 *Step 5*. Termination determination: When the decision-maker satisfies with the obtained solution *G*_*i*_(*ξ*^*k*^,  *a*_*i*_^*k*^), then stop the process with $\left(\bar{\xi }=\xi^{k},{\bar{a}}_{i}=a_{i}^{k}\right)$ as the final solution. On the other hand, when decision-maker is not satisfied with *G*_*i*_(*ξ*^*k*^,  *a*_*i*_^*k*^) and $G_{i}\left(\xi^{k},\;a_{i}^{k}\right)={\bar{G}}_{i}^{k}$ or *k*=*m*, then there is no satisfactory *α*−possibly efficient reference solution of *α* − MONLP. In that case, we proceed to Step 6.
Step 6. Modification of reference point by DM is as follows:
  (a) DM chooses any *f*_*k*_ in *I*^*k*^ such that *G*_*if*_*k*__ is an unsatisfactory objective in {*G*_*i*_ : *i* ∈ *I*^*k*^} at (*ξ*^*k*^,  *a*_*i*_^*k*^). Let *I*^*k*+1^=*I*^*k*^/{*f*_*k*_}.
  Separate *I*^*k*+1^ into the following two parts:
    (23) $$\begin{array}{c} I_{1}^{k}=\left\{\begin{array}{c} i\in {\text{I}}^{\text{k}+1}:\;{\text{G}}_{\text{i}}\left(\xi^{\text{k}},{\text{a}}_{i}^{\text{k}}\right)<{\bar{\text{G}}}_{i}^{\text{k}}\\ \text{and DM wishes to realise the value of }{\text{G}}_{\text{i}}\text{ at }{\text{G}}_{\text{i}}\left(\xi^{\text{k}},{\text{ a}}_{i}^{\text{k}}\right) \end{array}\right\}, \end{array}$$
    (24) $$\begin{array}{c} I_{2}^{k}=\frac{I^{k+1}}{I_{1}^{k}}. \end{array}$$
  (b) For *i* ∈ *I*_1_^*k*^, the decision-maker provides *Η*_*i*_^*k*^, be the amount to be relaxed for *G*_*i*_, such that ${Η}_{i}^{k}\in ]0,{\bar{G}}_{i}^{k}-G_{i}\left(\xi^{k},a_{i}^{k}\right)\;]$.
  Let ${\bar{\text{G}}}_{\text{i}}^{\text{k}+1}=G_{i}\left(\xi^{k},a_{i}^{k}\right)+{Η}_{i}^{k}$.
  For *i* ∈ *I*_2_^*k*^, let ${\bar{G}}_{i}^{k+1}=G_{i}\left(\xi^{k},a_{i}^{k}\right)$.
  For *i* ∈ *I*^*k*^/*I*^*k*+1^, let ${\bar{G}}_{i}^{k+1}={\bar{G}}_{i}^{k}.$
  (c) In the case of ${\bar{G}}_{i}^{k+1}=G_{i}\left(\xi^{k},a_{i}^{k}\right);\forall \;i\in I^{k}/\left\{f_{k}\right\}$, return to (a) to separate *I*^*k*+1^ again or to raise the amount to be relaxed for some *G*_*i*_,  *i* ∈ *I*_1_^*k*^ at G_i_(*ξ*^k^, a_*i*_^k^), go to (b) if the DM wishes to do that. Otherwise, stop and there is no satisfactory *α*−possibly optimal solution. In this case, we have ${\bar{\text{G}}}_{\text{i}}^{\text{k}+1}\neq G_{i}\left(\xi^{k},a_{i}^{k}\right)$, for some *i* ∈ *I*^*k*^/{*f*_*k*_}, go to (d).
  (d) Let $f=f_{k},{\bar{\text{G}}}_{\text{i}}^{\text{k}+1}=G_{i}^{\prime },i=1,2,\ldots ,n;i\neq f_{k}$, and solve the auxiliary problem (AP) as follows (AP) min*G*_*f*_(*ξ*, *a*_*f*_):
    (25) $$\begin{array}{c} G_{i}\left(\xi ,a_{i}\right)\leq G_{i}^{\prime },i=1,2,\ldots ,m;i\neq f, \end{array}$$
    (26) $$\begin{array}{c} \xi \in \Psi =\left\{\xi \in {\mathbb{R}}^{t}:{\dddot{h}}_{l}\left(\xi \right)\geq 0,l=1,2,\ldots ,s\right\}, \end{array}$$
    (27) $$\begin{array}{c} a_{i}\in u_{\alpha }\left({\tilde{a}}_{i}\right),i=\bar{1,m}. \end{array}$$
  Let (*ξ*^′*k*^, *a*^;*k*^) be the satisfactory *α*−possibly optimal solution.
  When *G*_*f*_*k*__(*ξ*^′*k*^, *a*_*f*_*k*__^′*k*^)=*G*_*f*_*k*__(*ξ*^*k*^, *a*_*f*_*k*__^*k*^) or *G*_*f*_*k*__(*ξ*^′*k*^, *a*_*f*_*k*__^′*k*^) for objective *G*_*f*_*k*__ is not satisfactory to the DM, return to (b) to increase the amount to be relaxed for some *G*_*i*_, *i* ∈ *I*_1_^*k*^ at *G*_*i*_(*ξ*^*k*^, *a*_*i*_^*k*^) if the DM wishes; otherwise, stop and there is no satisfactory *α*−possibly optimal solution.
  When *G*_*f*_*k*__(*ξ*^′*k*^, *a*_*f*_*k*__^′*k*^) ≠ *G*_*f*_*k*__(*ξ*^*k*^, *a*_*f*_*k*__^*k*^) and *G*_*f*_*k*__(*ξ*^′*k*^, *a*_*f*_*k*__^′*k*^) is satisfactory to the DM for objective *G*_*f*_*k*__, the DM provides *Η*_*f*_*k*__^*k*^, the largest amount to be improved for *G*_*f*_*k*__ such that Η_*f*_*k*__^*k*^ ∈ ]0, *G*_*f*_*k*__(*ξ*^*k*^, *a*_*f*_*k*__^*k*^) − *G*_*f*_*k*__(*ξ*^′*k*^, *a*_*f*_*k*__^′*k*^)].
  Let ${\bar{G}}_{f_{k}}^{k+1}=G_{f_{k}}\left(\xi^{k},a_{f_{k}}^{k}\right)-{Η}_{f_{k}}^{k}$.
  (e) If ${\bar{\text{G}}}_{{\text{f}}_{k}}^{\text{k}+1}=G_{f_{k}}\left(\xi^{\prime k},a_{f_{k}}^{\prime k}\right)$, let *k*=*k*+1 and return to step (c). Otherwise, let (*ξ*^*k*+1^, *a*_*i*_^*k*+1^)=(*ξ*^′*k*^, *a*_*i*_^′*k*^), *k*=*k*+1, and return to (d) when (*ξ*^′*k*^, *a*_*i*_^′*k*^) is a unique *α*−possibly optimal solution of (AP) or let (*ξ*^′*k*^, *a*_*i*_^′*k*^) be an *α*−possibly optimal solution of the following problem:
    (28) $$\begin{array}{c} \underset{i=1,\;2,\ldots ,\;m}{\min}\gamma_{i}, \end{array}$$
    (29) $$\begin{array}{c} {\bar{y}}_{i}\left|G_{i}\left(\xi ,a_{i}\right)-{\overset{ˇ}{G}}_{i}\right|\leq \gamma_{I},i=1,2,\ldots ,m, \end{array}$$
    (30) $$\begin{array}{c} \xi \in \Psi =\left\{\xi \in {\mathbb{R}}^{t}:{\dddot{h}}_{l}\left(\xi \right)\geq 0,\;l=1,2,\ldots ,\;s\right\} \end{array}$$
Let *k*=*k*+1, and return to (c). If ${\bar{\text{G}}}_{{\text{f}}_{k}}^{\text{k}+1}\geq {\text{G}}_{{\text{f}}_{\text{k}}}\left(\xi^{\text{'k}},{\text{a}}_{{\text{f}}_{\text{k}}}^{\text{'k}}\right)$, put k=k+1, and return to Step 4.
Step 7. Determine the first-kind stability set $S\left(\bar{\xi },\;\bar{a_{i}}\right)$ by applying the following conditions:

(31) $$\begin{array}{c} \sigma_{i}\left({\bar{a}}_{i}-d_{2i}\right)=0,i=\bar{1,m}, \end{array}$$

(32) $$\begin{array}{c} \rho_{i}\left(d_{1i}-{\bar{a}}_{i}\right)=0,\bar{1,m}, \end{array}$$

(33) $$\begin{array}{c} \sigma_{i},\rho_{i}\geq 0,d_{1i},d_{2i}\in \mathbb{R},\left[d_{1i},d_{2i}\right]=u_{\alpha }\left({\tilde{a}}_{i}\right)\bar{1,m}. \end{array}$$

**Figure 2:**
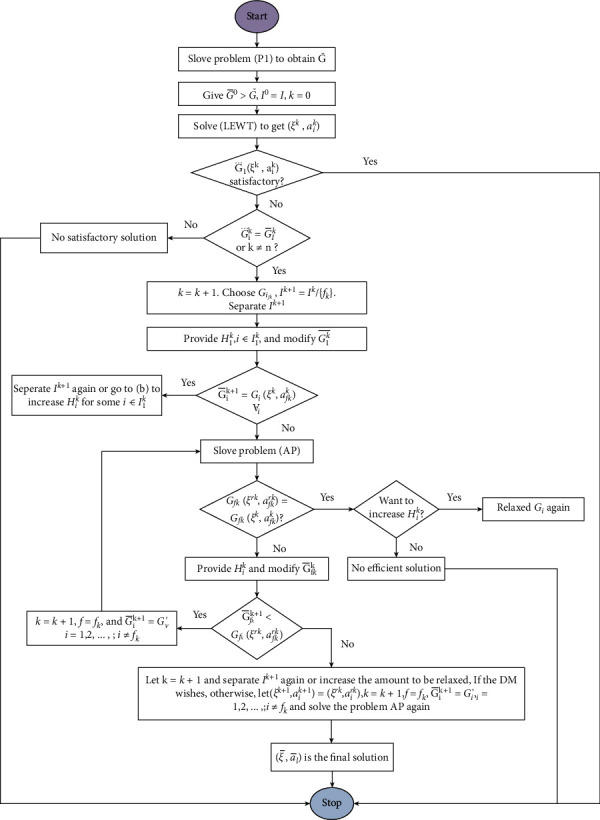
Flow chart of the proposed approach.

All the abovementioned steps are summarized through a flowchart given in Figure 2.

## 5. Numerical Example

In this section, the approach mentioned above is illustrated with a numerical example.

Consider the following two-player game with

(34) $$\begin{array}{c} G_{1}\left(\xi ,{\tilde{a}}_{1}\right)={\left(\xi_{1}-{\tilde{a}}_{1}\right)}^{2}+{\left(\xi_{2}-1\right)}^{2}, \end{array}$$

(35) $$\begin{array}{c} G_{2}\left(\xi ,{\tilde{a}}_{2}\right)={\left(\xi_{1}-1\right)}^{2}+{\tilde{a}}_{2}{\left(\xi_{2}-2\right)}^{2}, \end{array}$$

where player I controls *ξ*_1_ ∈ ℝ and player II controls *ξ*_2_ ∈ ℝ with the constraints as follows:

(36) $$\begin{array}{c} 0\leq \xi_{1}\leq 4;0\leq \xi_{2}\leq 4. \end{array}$$

**Figure 3:**
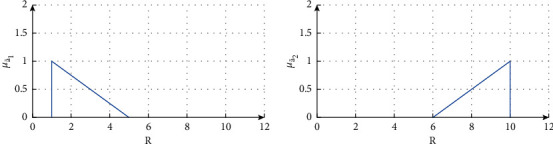
Possibility distributions (a) $\mu_{{\tilde{a}}_{1}}\left(.\right)$ for ${\tilde{a}}_{1}$ and (b) $\mu_{{\tilde{a}}_{2}}\left(.\right)$ for ${\tilde{a}}_{2}$.

The possibilistic variables ${\tilde{a}}_{1}$ and ${\tilde{a}}_{2}$ are characterized by a possibility distribution $\mu_{{\tilde{a}}_{1}}\left(.\right)$ and $\mu_{{\tilde{a}}_{2}}\left(.\right)$ as mentioned in Figure 3. The supports of the possibilistic variables ${\tilde{a}}_{1}$ and ${\tilde{a}}_{2}$ are taken as [1,  5] and [6,10], respectively. Hence, for the parametric function 0 ≤ *ϑ* ≤ 1, the supports are stated as

(37) $$\begin{array}{c} \text{Supp }\left({\tilde{\text{a}}}_{1}\right)=1+4\vartheta ,\mu_{{\tilde{\text{a}}}_{1}}\left(1\right)=\mu_{{\tilde{\text{a}}}_{1}}\left(5\right)=0,\\ \text{Supp }\left({\tilde{\text{a}}}_{2}\right)=10-4\vartheta ,\mu_{{\tilde{\text{a}}}_{2}}\left(10\right)=\mu_{{\tilde{\text{a}}}_{2}}\left(6\right)=0. \end{array}$$

Then, the steps of the proposed approach are illustrated as follows:

*Step 1*. Without loss of generality, consider the value of *α*=0.4. With this value, we formulate the *α* − PMONLP model as
  (38) $$\begin{array}{c} \min\left(\begin{array}{c} G_{1}\left(\xi ,a_{1}\right)={\left(\xi_{1}-1-4\vartheta \right)}^{2}+{\left(\xi_{2}-1\right)}^{2},\\ G_{2}\left(\xi ,a_{2}\right)={\left(\xi_{1}-1\right)}^{2}+\left(10-4\vartheta \right){\left(\xi_{2}-2\right)}^{2} \end{array}\right), \end{array}$$
subject to *ξ*_1_ − 4 ≤ 0, *ξ*_2_ − 4 ≤ 0, *ξ*_1_, *ξ*_2_ ≥ 0,; *ϑ* ∈ [0,1].
*Step 2*. The equivalent crisp optimization model of the above model is given as
  (39) $$\begin{array}{c} \underset{i=1,\;2}{\max}w_{i}, \end{array}$$
  (40) $$\begin{array}{c} {\left(\xi_{1}-1-4\vartheta \right)}^{2}+{\left(\xi_{2}-1\right)}^{2}\geq w_{1}, \end{array}$$
  (41) $$\begin{array}{c} {\left(\xi_{1}-1\right)}^{2}+\left(10-4\vartheta \right){\left(\xi_{2}-2\right)}^{2}\geq w_{2}, \end{array}$$
  (42) $$\begin{array}{c} \xi_{1}-4\leq 0,\xi_{2}-4\leq 0,\xi_{1},\xi_{2}\geq 0,;\vartheta \in \left[0,1\right], \end{array}$$
  (43) $$\begin{array}{c} w_{i}\in \mathbb{R}. \end{array}$$
After solving this model, we get the optimal decision variables as
  (44) $$\begin{array}{c} \left({\overset{ˇ}{\xi }}_{1},{\overset{ˇ}{\xi }}_{2},{\overset{ˇ}{a}}_{1},{\overset{ˇ}{a}}_{2}\right)=\left(3.9989,3.9996,1.0028,9.9972\right). \end{array}$$
And hence $\overset{ˇ}{G}={\left(16.390,48,9662\right)}^{T}$.
*Step 3*. Assume the decision-maker provides the initial reference point ${\bar{G}}^{0}$ such that ${\bar{G}}^{0}>\overset{ˇ}{G}.$ As $\overset{ˇ}{G}={\left(16.390,48,9662\right)}^{T}$, consider the initial reference point as ${\bar{G}}^{0}={\left(48,55\right)}^{T}$. Set  *I*^0^=*I*,  *k*=0.
*Step 4*. By taking ${\bar{y}}_{i}={\bar{G}}_{i}^{k}-{\overset{ˇ}{G}}_{i}/\sum_{i=1}^{n}\left({\bar{G}}_{i}^{k}-{\overset{ˇ}{G}}_{i}\right)\text{for }i=1,2,$ we get ${\bar{y}}_{1}=0.8397\text{ and }{\bar{y}}_{2}=0.16029$.
Hence, we formulate the LWTP model as
  (45) $$\begin{array}{c} \text{Lex }\underset{\text{i}=\text{i},\;2}{\min}\left\{\begin{array}{c} \gamma_{\text{i}},\;\\ \left|{\left(\xi_{1}-1-4\vartheta \right)}^{2}+{\left(\xi_{2}-1\right)}^{2}-16.390\right|,\\ \left|{\left(\xi_{1}-1\right)}^{2}+\left(10-4\vartheta \right){\left(\xi_{2}-2\right)}^{2}-48.9662\right| \end{array}\right\}\\ 0.8397\left|{\left(\xi_{1}-1-4\vartheta \right)}^{2}+{\left(\xi_{2}-1\right)}^{2}-16.390\right|\leq \gamma_{\text{I}},\\ 0.16029\left|{\left(\xi_{1}-1\right)}^{2}+\left(10-4\vartheta \right){\left(\xi_{2}-2\right)}^{2}-48.9662\right|\leq \gamma_{2};\\ \xi_{1}-4\leq 0,\xi_{2}-4\leq 0,\xi_{1},\xi_{2}\geq 0,;\vartheta \in \left[0,1\right];0\leq \gamma_{\text{i}}\in \mathbb{R} \end{array}$$
The optimal solution of this model is obtained as $\left({\bar{\xi }}_{1},{\bar{\xi }}_{2},{\bar{a}}_{1},{\bar{a}}_{2}\right)={\left(3.15562,3.413545,4.36004,6.63996\right)}^{T}$ and hence $G\left({\bar{\xi }}_{1},{\bar{\xi }}_{2},{\bar{a}}_{1},{\bar{\;a}}_{2}\right)={\left(4.62078,7.93297\right)}^{T}$.
*Step 5*. From the above solutions, we get after the first iteration as follows:
  (46) $$\begin{array}{c} \left({\bar{\xi }}_{1},{\bar{\xi }}_{2},{\bar{a}}_{1},{\bar{\;a}}_{2}\right)={\left(3.15562,3.413545,4.36004,6.63996\;\right)}^{T}\\ G\left({\bar{\xi }}_{1},{\bar{\xi }}_{2},{\bar{a}}_{1},{\bar{\;a}}_{2}\right)={\left(4.62078,7.93297\right)}^{T}, \end{array}$$
and the reference point ${\bar{\text{G}}}^{0}={\left(48,55\right)}^{T}$ and $\overset{ˇ}{G}={\left(16.390,48,9662\right)}^{T}.$ Is the decision-maker satisfying with the solution: Y/N?
Assume that an expert is satisfied with such solution and hence go to Step 7 directly.
*Step 7*. Now, the first-kind stability set is determined.

For the solution set, *S*(3.15562, 3.413545, 4.36004, 6.63996), the first-kind stability set is determined by applying the following conditions:

(47) $$\begin{array}{c} \sigma_{1}\left(4.36004-d_{21}\right)=0,\\ \sigma_{2}\left(6.63996-d_{22}\right)=0,\\ \sigma_{1},\sigma_{2}\geq 0. \end{array}$$

For *J*_1_⊆{1,2}, *J*_1_=∅, *σ*_1_, *σ*_2_=0. Then,

(48) $$\begin{array}{c} S_{J_{1}}\left(\begin{array}{c} 3.15562,3.413545,\\ 4.36004,6.63996 \end{array}\right)=\left\{\begin{array}{c} d_{2}\in {\mathbb{R}}^{2}:\;d_{21}\geq 4.36004,\\ d_{22}\geq 6.63996\; \end{array}\right\}. \end{array}$$

For *J*_2_={1}, *σ*_1_ > 0, *σ*_2_ > 0. Then,

(49) $$\begin{array}{c} S_{J_{2}}\left(\begin{array}{c} 3.15562,3.413545,\\ 4.36004,6.63996 \end{array}\right)=\left\{\begin{array}{c} d_{2}\in {\mathbb{R}}^{2}:\;d_{21}=4.36004,\\ d_{22}\geq 6.63996\; \end{array}\right\}. \end{array}$$

For *J*_3_={2}, *σ*_1_=0, *σ*_2_ > 0. Then,

(50) $$\begin{array}{c} S_{J_{3}}\left(\begin{array}{c} 3.15562,3.413545,\\ 4.36004,6.63996 \end{array}\right)=\left\{\begin{array}{c} d_{2}\in {\mathbb{R}}^{2}:\;d_{21}\geq 4.36004,\\ d_{22}=6.63996\; \end{array}\right\}. \end{array}$$

For *J*_4_={12}, *σ*_1_ > 0, *σ*_2_ > 0. Then,

(51) $$\begin{array}{c} S_{J_{4}}\left(\begin{array}{c} 3.15562,3.413545,\\ 4.36004,6.63996 \end{array}\right)=\left\{\begin{array}{c} d_{2}\in {\mathbb{R}}^{2}:d_{21}=4.36004,\\ d_{22}=6.63996\; \end{array}\right\}. \end{array}$$

(52) $$\begin{array}{c} S\left(\begin{array}{c} 3.15562,3.413545,\\ 4.36004,6.63996 \end{array}\right)=\sum_{q=1}^{4}S_{J_{q}}\left(\begin{array}{c} 3.15562,3.413545,\\ 4.36004,6.63996 \end{array}\right). \end{array}$$

## 6. Characteristic Comparison

In this section, the proposed approach has been compared with some existing literatures [23–27] in terms of their characteristic features to illustrate the advantages of the suggested approach. The results for this analysis are summarized in Table 1. The symbol “✗” or “✓” shown in the table represents whether the associated feature satisfy or not. Also, it is mentioned that the proposed approach has considered the environment of uncertainty and possibility while all the others have taken either the fuzzy or crisp environment to solve the game problems.

It is also seen from the table that the proposed method utilizes the Lexicographic weighted Tchebycheff model to compute the weights. In contrast, all other existing models fail to deal with it. Other than that the method proposed in [24, 26, 27] also derived the efficient solution for the problem along with the proposed one, but all these existing methods have considered the uncertainties with fuzzy variables; however, in the proposed method, a possibility variable has been used to address the uncertainties. The proposed method suggested the interactive approach based on the decision-maker preferences. Utilizing this feature, an expert can change their preferences if not satisfied with the obtained result through the process. Through it, a person can select the desired one as per their choices related to optimism or pessimism towards the objective of the problem.

## 7. Conclusion

The main contribution of the paper can be summarized as follows:

1. In this study, we presented a novel idea related to the continuous cooperative static game (CCSTG) with possibilistic parameters associated with the cost functions of the *m* players. To handle the uncertainties in the model and a conflicting nature between the objectives, a possibilistic variable ${\tilde{a}}_{i}$ is taken instead of fuzzy variable, which is categorized by a possibilistic distribution $\mu_{{\tilde{a}}_{i}}:V⟶\left[0,1\right]$ for *i*=1,2,…, *n*.
2. By utilizing the concept of *α*−cut, the considered Poss-CCSTG model is converted into the crisp *α*-CCSTG problem and hence into the *α* − PMONLP model.
3. The relationship between the *α*−efficient solution of the *α*-CCSTG problem and *α* − PMONLP model is derived. From the study, we conclude that *ξ*^*∗*^ ∈ Ψ is an *α*−possibly efficient solution of the *α*-CCSTG problem if and only if *ξ*^*∗*^ ∈ Ψ is an *α*−parametric efficient solution for *α* − PMONLP.
4. An interactive approach has been presented in the study to solve the obtained model. The significant advantages of the proposed model are that it is a generic method that applies to any convex or nonconvex, differentiable, or nondifferentiable problem. Another advantage is that in the proposed interactive approach, there is no need to provide the reference set (feasible or infeasible) in advance by the decision-maker to solve the problem. In addition to it, a set of the first kind, which corresponds to the stability set of the final solution, is determined.
5. Based on the solution obtained through the approach, a decision-maker can analyze the impact of the different objectives as per their desired goals. If the decision-maker is satisfied with the output, they can stop the algorithm and obtain the optimal results; otherwise, they will modify their preferences and improve the results that are met according to their goals and hence select their optimal design parameters.

In the future, the result of the presented approach shall be extended to some other real-life problems related to different optimization models. Also, we shall extend the proposed approach under the different uncertain environments such as possibility-Pythagorean and interval-valued Pythagorean fuzzy set, spherical fuzzy set, and intuitionistic fuzzy set [34–37].

## Figures and Tables

**Table 1:** Characteristic comparison of the proposed method with other existing methods.

Ref.	Features				Environment
	Interactive approach	Continuous cooperative static games	Best compromise solution	Lexicographic weighted Tchebycheff program	
Zaichnko et al. [24]	✗	✓	✗	✗	Uncertainty/fuzzy
Donahue et al. [25]	✗	✗	✓	✗	Deterministic/certain
Zhou et al. [26]	✗	✓	✗	✗	Uncertainty/fuzzy
Khalifa and Kumar [23]	✗	✓	✗	✗	Deterministic/certain
Khalifa et al. [27]	✗	✓	✗	✗	Uncertainty/fuzzy
Proposed study	✓	✓	✓	✓	Uncertainty/possibilistic

## Data Availability

No data were used to support this study

## References

[1] Taha H. A. (2019). *Operations Research: An Introduction”*.

[2] Vincent T. L., Grantham W. J. (1981). *Optimality in Parametric Systems*.

[3] Zadeh L. A. (1965). Fuzzy sets. *Information and Control*.

[4] Dubois D., Prade H. (1980). *Fuzzy Sets and Systems; Theory and Applications*.

[5] Bellman R. E., Zadeh L. (1970). Decision-making in a fuzzy environment. *Management Science*.

[6] Kaufmann A., Gupta M. M. (1988). *Fuzzy Mathematical Models in Engineering and Management Science*.

[7] Ebrahimnejad A. (2019). An effective computational attempt for solving fully fuzzy linear programming using MOLP problem. *Journal of Industrial and Production Engineering*.

[8] Osman M. S. A. Different parametric problems in continuous static games. *The First ORMA Conference*.

[9] Osman M. S. A., El- Banna A. H., Kamel M. M. (1999). On fuzzy continuous static games (FCSG) stackelberg leader with min- max followers. *Journal of Fuzzy Mathematics*.

[10] Osman M. S. A., El- Bannna A., Amer A. H. (1999). Study on large-scale fuzzy Nash equilibrium solutions. *Journal of Fuzzy Mathematics*.

[11] Elshafei M. M. K. (2007). An interactive approach for solving Nash cooperative continuous static games (NCCSG). *International Journal of Contemporary Mathematical Sciences*.

[12] Cruz J. B., Simaan M. A. (2000). Ordinal games and generalized Nash and stackelberg solutions. *Journal of Optimization Theory and Applications*.

[13] Kacher F., Larbani M. (2008). Existence of equilibrium solution for a non-cooperative game with fuzzy goals and parameters. *Fuzzy Sets and Systems*.

[14] A.Khalifa H., ZeinEldin R. A. (2015). An interactive approach for solving fuzzy cooperative continuous static games. *International Journal of Computer Application*.

[15] Navidi H., Amiri A., Kamranrad R. (2014). Multi- responses optimization through game theory approach. *International Journal of Industrial Engineering and Production static games*.

[16] Corley H. W. (2015). A mixed cooperative dual to the Nash equilibrium. *Game Theory*.

[17] Farooqui A. D., Niazi M. A. (2016). Game theory models for communication between agents: a review. *Complex Adaptive Systems Modeling*.

[18] Sasikala G., Kumaraghuru S. (2017). Interactive decision-making approach for Nash cooperative continuous static games. *International Journal of Recent Scientific Research*.

[19] Khalifa H. A. (2018). An interactive approach for solving multi- objective nonlinear programming and its application on cooperative continuous static games. *Journal of Applied Research on Industrial Engineering*.

[20] Ebrahimnejad A. (2015). A duality approach for solving bounded linear programming problems with fuzzy variables based on ranking functions and its application in bounded transportation problems. *International Journal of Systems Science*.

[21] Silbermayr L. (2020). A review of non-cooperative newsvendor games with horizontal inventory interactions. *Omega*.

[22] Shuler R. L. (2019). Wealth-relative effects in cooperation games. *Heliyon*.

[23] Wahed Khalifa H. A. E., Kumar P. (2020). Multi-objective optimization for solving cooperative continuous static games using Karush. *International Journal of Operational Research*.

[24] Zaichenko H. (2020). Fuzzy cooperative games of two players under uncertainty conditions. *2020 IEEE 2nd International Conference on System Analysis & Intelligent Computing (SAIC)*.

[25] Donahue K., Hauser O. P., Nowak M. A., Hilbe C. (2020). Evolving cooperation in multichannel games. *Nature Communications*.

[26] Zhou J., Tur A., Petrosian O., Gao H. (2021). Transferable utility cooperative differential games with continuous updating using pontryagin maximum principle. *Mathematics*.

[27] Khalifa H. A., Alodhaibi S. S., Saeed M., Rahman A. U. A. (2022). A Study on cooperative continuous static games without differentiability under fuzzy environment. *International Journal of Fuzzy System Applications*.

[28] Petrosyan L., Yeung D. (2021). Shapley value for differential network games: theory and Application. *Journal of Dynamics and Games*.

[29] Wang Z., Petrosian O., Petrosian O. (2020). On class of non-transferable utility cooperative differential games with continuous updating. *Journal of Dynamics and Games*.

[30] Hussein M. L. (1992). On convex vector optimization problems with possibilistic weights. *Fuzzy Sets and Systems*.

[31] Abd El-Hady Kassem M. (1998). Stability of possibilistic multiobjective nonlinear programming problems without differentiability. *Fuzzy Sets and Systems*.

[32] Luhandjula M. K. (1986). On possibilistic linear programming. *Fuzzy Sets and Systems*.

[33] Luhandjula M. K. (1987). Multiple objective programming problems with possibilistic coefficients. *Fuzzy Sets and Systems*.

[34] Ebrahimnejad A., Verdegay J. L. (2016). An efficient computational approach for solving type-2 intuitionistic fuzzy numbers based Transportation Problems. *International Journal of Computational Intelligence Systems*.

[35] Wang X. M., Qin Z. L., Hu Y. D. (2001). An interactive algorithm for multicriteria decision making: the attainable reference point method. *IEEE Transactions on Systems, Man, and Cybernetics - Part A: Systems and Humans*.

[36] Yu Q., Liu X., Qin J., Zhou L., Garg H., Mardani A. (2022). Consensus reaching for prospect cross-efficiency in data envelopment analysis with minimum adjustments. *Computers & Industrial Engineering*.

[37] Yang Z., Shang W.-L., Zhang H., Garg H., Han C. (2022). Assessing the green distribution transformer manufacturing process using a cloud-based q-rung orthopair fuzzy multi-criteria framework. *Applied Energy*.

